# Dynamic distribution of *Massilia* spp. in sewage, substrate, plant rhizosphere/phyllosphere and air of constructed wetland ecosystem

**DOI:** 10.3389/fmicb.2023.1211649

**Published:** 2023-07-27

**Authors:** Ailing Xu, Congcong Liu, Shuke Zhao, Zhiwen Song, Hui Sun

**Affiliations:** ^1^School of Environmental and Municipal Engineering, Qingdao Technological University, Qingdao, Shandong, China; ^2^Qingdao sub-Center, Shandong Water Transfer Project Operation and Maintenance Center, Qingdao, Shandong, China

**Keywords:** *Massilia bacterial*, clonal library, distribution characteristics, community structure, 16S rDNA extraction

## Abstract

**Introduction:**

*Massilia* bacteria are widely distributed and have various ecological functions. Preliminary studies have shown that *Massilia* is the dominant species in constructed wetland ecosystems, but its species composition and distribution in constructed wetlands are still unclear.

**Methods:**

In this paper, the in-house-designed primers were used to construct a 16S rDNA clone library of *Massilia*. The RFLP sequence analysis method was used to analyze the diversity of *Massilia* clone library and the composition of *Massilia* in sewage, substrate, plant rhizosphere, plant phyllosphere and air in a constructed wetland sewage treatment system. Redundancy analysis (RDA) and canonical correspondence analysis (CCA) were used to analyze the correlation between environmental factors and the population characteristics of *Massilia* in the corresponding environment. The dominant species of *Massilia* were analyzed for differences.

**Results:**

The results showed that the 16S rDNA clone library in primer 5 worked well. According to the clone library diversity index analysis, the richness of *Massilia* varied significantly in different environments in different seasons, where the overall summer and autumn richness was higher than that in the spring and winter. The relative abundance of 5 *Massilia* in the constructed wetland ecosystem was greater than 1% in all samples, which were *M. alkalitolerans*, *M. albidiflava*, *M. aurea*, *M. brevitalea*, and *M. timonae*. The seasonal variation of dominant genera was significantly correlated with environmental factors in constructed wetlands.

**Discussion:**

The above results indicated that the species of *Massilia* were abundant and widely distributed in the constructed wetland ecosystem, and there were significant seasonal differences. In addition, the *Massilia* clone library of constructed wetland was constructed for the first time in this study and the valuable data of *Massilia* community structure were provided, which was conducive to the further study of microbial community in constructed wetland.

## 1. Introduction

*Massilia* spp. are Gram-negative bacteria that are widely distributed. For example, four species of *Massilia* were isolated from Antarctic streams, lakes and weathering layers (Holochová et al., 2020). *Massilia aquatica* was isolated from a subtropical stream in China (Lu et al., 2020). *Massilia* sp. YMA4 was isolated from the open ocean off Lamay island in Taiwan (Ho et al., 2021), and *M. buxea* was found on rock surfaces (Sun et al., 2017). In addition, *Massilia* spp. can also be found in water, organisms and human clinical specimens. Among those, soil is the main environment of *Massilia* spp (Hye et al., 2022).

Numerous studies have shown that *Massilia* spp. have many functions. Some *Massilia* are able to synthesize violacein (Agematu et al., 2011; Baek et al., 2014), which has a good therapeutic effect on tumors, malaria and diarrhea and has great application potential in the pharmaceutical field. PHAs (as reserve material displayed as intracellular bright granules) is a kind of natural polymer biomaterial, which has good biocompatibility and biodegradability, and has become the most active research focus in the field of biomaterials in recent years. For the first time, Cerrone et al. find majority of the *Massilia* strains were capable to produce significant amounts of PHA under batch culture mode using soluble starch as carbon source (Cerrone et al., 2011). Studies have shown that some *Massilia* spp. were beneficial to environmental modification. For example, *M. aromaticvorans* ML15P13^T^ can degrade toxic gaseous organic air pollutants, such as benzene, toluene, ethylbenzene and xylene isomers (m, p and o-x) (BTEX) (Son et al., 2021); *M. tieshanensis* TS3 and *M. putida* 6NM-7 are resistant to multiple heavy metals (Du et al., 2012; Feng et al., 2016); *M. phosphatilytica* 12-OD1 has phosphate solubilization and can improve available phosphorus content in soil and improve the soil environment (Zheng et al., 2017); *Massilia* sp. WF1 and *Massilia* sp. WG5 can degrade phenanthrene and repair contaminated soil (Lou et al., 2016; Gu et al., 2021); and *M. chloroacetimidivorans* TA-C7e degrades the chloroacetamide herbicide (Lee et al., 2017). Some *Massilia* bacteria can also promote plant growth and increase the survival rate of plants in harsh environments. Krishnamoorthy et al. found that the combined application of *Massilia* and arbuscular mycorrhizal fungi alleviated the effects of salt stress on plant growth, root colonization, nutrient accumulation and growth in studying the effects of three different salinity on the growth of corn plants in coastal reclamation area (Krishnamoorthy et al., 2016). Turnbull et al. isolated a strain of *Massilia* from potato roots that can produce heteroauxin and cellulose-degrading enzymes to promote plant growth (Turnbull et al., 2012).

In recent years, research on *Massilia* spp. made significant progress. However, the community composition and distribution of *Massilia* spp. in the constructed wetland sewage treatment system has been neglected. Preliminary research on this research group showed that *Massilia* spp. are the dominant bacteria in the air of constructed wetland sewage treatment systems. The pollutant degradation ability of artificial wetland sewage treatment systems is higher than that in natural wetlands and microbes play an important role (Liu et al., 2019; Hu et al., 2022). In consideration of the functions of *Massilia* spp., it is of great significance to study the community composition of *Massilia* bacteria in constructed wetland ecosystems. The culturable microorganism accounts for 1% ~ 5%; therefore, molecular biology has been widely used in microbial community research. Lang et al. and Zhang et al. studied the community structure of constructed wetlands using molecular biology methods (Zhang et al., 2019; Lang et al., 2021). This study was the first to investigate the composition and distribution characteristics of *Massilia* in sewage, substrate, plant rhizosphere, plant phyllosphere and air in a constructed wetland sewage treatment system by two molecular biology methods: construction of a 16S rDNA clone library and RFLP sequence analysis. At the same time, redundancy analysis (RDA) and canonical correspondence analysis (CCA) were used to analyze the correlation between environmental factors and population characteristics of *Masilia*. The compositional differences and dynamic changes of *Massilia* in different environments and seasons were also explored, which is of great significance to reveal the mechanism of degradation of pollutants in the constructed wetlands and is beneficial to the operation of constructed wetlands.

## 2. Materials and methods

### 2.1. Sampling sites

**Figure 1:**
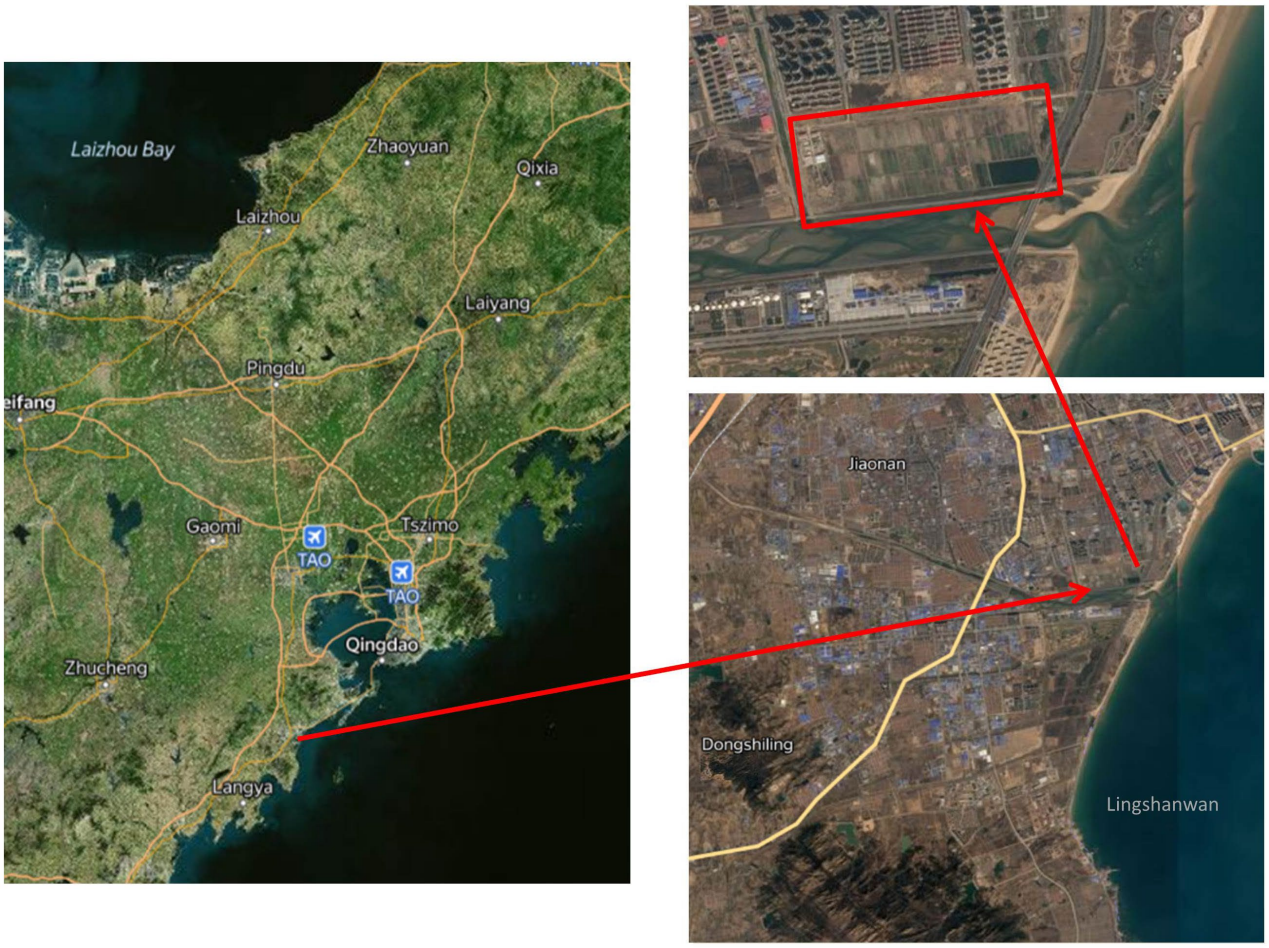
Schematic diagram of the geographic location of the study area.

The free water surface constructed wetland, located in the interior of the Huangdao district (Qingdao city, Shandong province, China), at a latitude of 35°35′ to 36°08’ North and a longitude of 119°30′ to 120°11′ East, is a part of an integrated sewage purification system (Figure 1). This region has a warm temperate continental monsoon climate with a mean annual temperature of 12°C and a mean annual precipitation of 794 mm. The constructed wetland wastewater treatment system had a total area of 76.7 hm^2^ and a treatment capability of 3.0 × 10^4^ m^3^·d^−1^ and was surrounded by the Yellow Sea to the east and south. It consisted of 99 treatment beds and received secondary unchlorinated wastewater from the Jiaonan Municipal Wastewater Treatment Facility with an A^2^O application as the secondary treatment. All beds were planted with common reed (*Phragmites australis*) and a number of naturally germinated wetland plants (*Typha orientalis*, *Scirpus validus*, and *Lemna minor*). To facilitate the harvest progress of aboveground biomass, sewage did not enter the constructed wetland bed from December to March of the following year.

**Figure 2:**
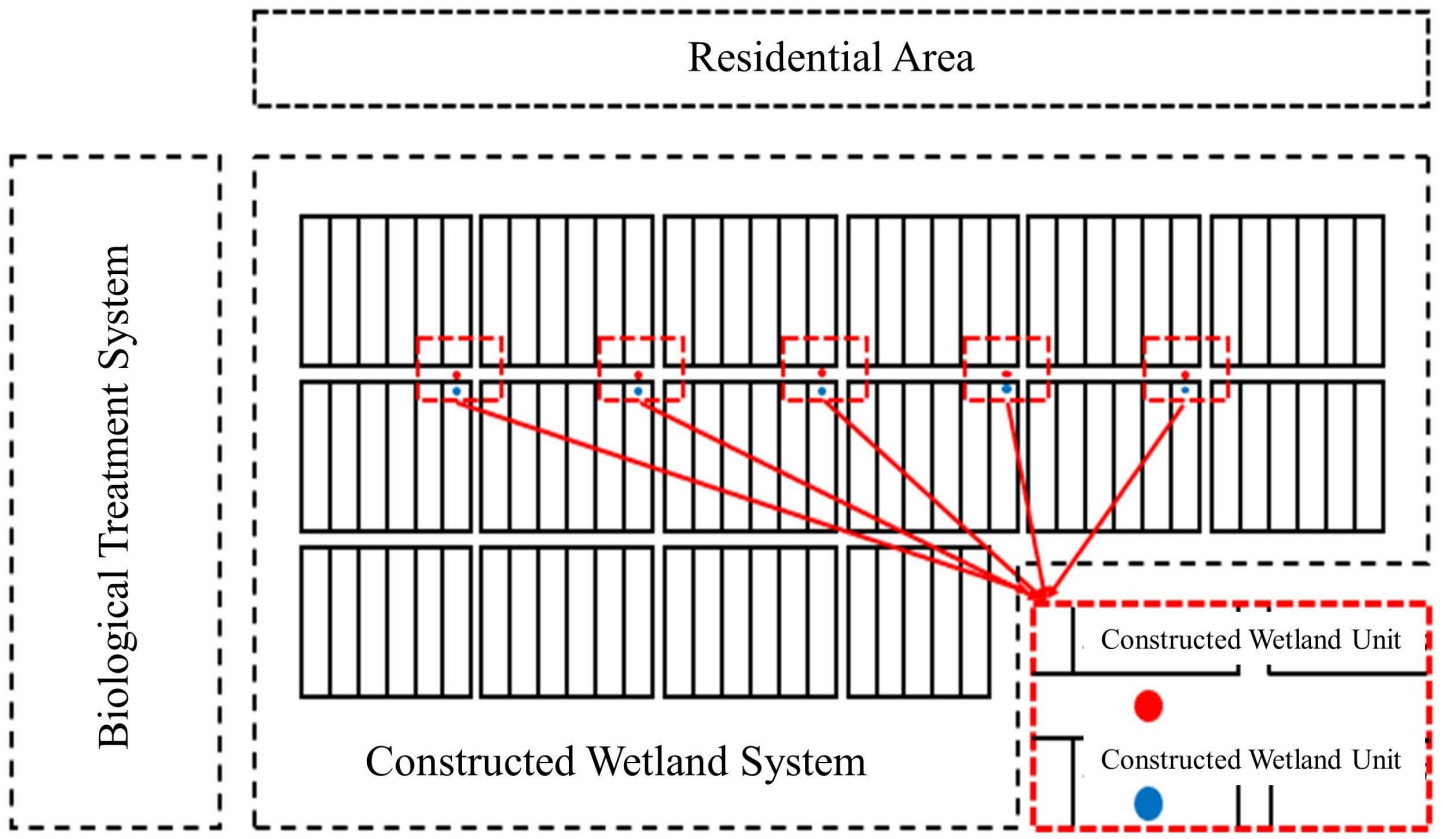
Schematic diagram of the sampling location of the constructed wetland. For the specific location of 5 sampling points, air samples, leaf interval, sewage, substrate and rhizome samples.

A total of five sampling points were selected in this experiment, as shown in Figure 2. The soil, wastewater, leaves and air were sampled from April 2018 to January 2019, and the sampling collection frequency was once every month.

### 2.2. Sampling method

Fifty grams of soil sample and 10 L of water sample were collected from each sample site using sterile sealed bags and sterile bottles, respectively. After removing the fine roots in the soil samples, the water and soil samples were transferred to the laboratory immediately. After samples were dewatered by centrifugation, a fraction of the soil samples was stored at −20°C for molecular analysis.

The rhizosphere samples of 3 reed plants were randomly selected at each sampling point. Large soil was removed from the roots, the roots were soaked in sterile water, and the difficult soil on the roots was rinsed with sterile water. The collected water samples were mixed evenly, concentrated by centrifugation and placed in a sterile centrifuge tube to extract DNA.

Leaves of the reed plants were aseptically collected from different locations within the experimental unit and placed in Ziploc bags or Whirl-Pak bags by using new gloves for each replicate and by applying an ethanol disinfection of the pruning shears between samples. Samples were then transported back to the laboratory at 4°C. One hundred milliliters of sterile water was added to the bags, and the samples were agitated for 1 min by hand and then sonicated for 2 min. The microfloral wash was then transferred to polypropylene tubes and centrifuged at 30,000 ×*g* overnight at 4°C. The pellet was then transferred to a microcentrifuge tube and stored at −80°C until DNA extraction was performed.

The bacterial activity samples in TSP were collected via two KC-1000 high-flow air samplers with fixed Teflon membranes. The samples were placed 1.5 m from the ground, and the TSP samples were collected on the membranes at a sampling flow rate of 1250 L/min. After 24 h of sample collection, the membranes of the samples were quickly sent back to the laboratory and stored at −80°C until subsequent analysis.

### 2.3. Experimental methods

#### 2.3.1. DNA extraction

Sterile distilled water was used to rinse the surface of the Teflon membrane to obtain the particulate matter. After centrifugation, the particulate matter was transferred to a sterile 2 mL centrifuge tube. The sample quality was maintained at 0.2 ~ 0.3 g. Using the E.Z.N.A. VR Soil DNA Kit (Omega Bio-Tek, USA), DNA samples were obtained according to the manufacturer’s instructions. An ultramicro spectrophotometer (IMPLEN, Germany) was used to detect the concentration.

#### 2.3.2. PCR amplification of the gene fragment

Five in-house-designed primers were amplified for 16S rDNA, and the community diversity of *Massilia* was analyzed by RFLP (Table 1). The PCR amplification procedure was as follows: 94°C for 5 min; 94°C for 30 s, annealing temperature Tm°C for 30 s, and 72°C for 1 min (36 cycles), and 72°C extension for 10 min.

**Table 1:** The primers used in the RFLP.

Number	Primers	Primer sequences (5′ - 3′)	Temperature/°C	Amplified fragment length/bp
1	Forward	GATAACGTAGCGAAAGTTACGCTAATAC	55	1606
	Reverse	CTCCTTGCGGTTAAGCTACCTACT		
2	Forward Reverse	TAGCAGAGTTCTGCGCAATCC GGCAGCACGGGCTTCGGCCTGGT	53	963
3	Forward Reverse	GGACGACCAGCCACACTGGGACTGAGACA TGGTAAAACCCGCTCCCATGGAGAGAG	58	1491
4	Forward Reverse	TAAGCTACCTACTTCTGGTAAAACCCA CCAAGAGTGGGGGATAACGTAGCGAAAGTT	52	1132
5	Forward Reverse	GACACGGTCCAGACTCCTAC TGTGAAGCCCTACCCATAA	53	903

Five pairs of primers were designed according to the 16S rRNA of *Massilia* spp. The PCR had a total volume of 25 μL, including 5 μL 5_reaction buffer, 5 μL 5_GC buffer, 2 μL dNTP (2.5 mmol L-1), 1 μL forward primer (10 lmol L-1), 1 μL reverse primer (10 lmol L-1), 2 μL DNA template, 8.75 μL ddH2O, and 0.25 μL Q5 DNA polymerase. The PCR system was initially denatured at 94°C for 5 min, followed by 29 cycles at 94°C for 30 s, 56°C for 30 s, and 72°C for 45 s, then extension at 72°C for 5 min, and finally samples were kept at 4°C. The Illumina MiSeq platform (Illumina, San Diego, CA, USA) provided by Personalbio (Shanghai, China) was selected for the sequencing of the PCR products.

#### 2.3.3. Construction of a 16S rDNA clone library and RFLP sequence analysis and sequencing

PCR products were purified by a PCR product Purification Kit (OMEGA). Purified PCR products were inserted into the T-vectors by a T4 ligase cloning kit (MBI Fermen-tas). The recombinant was transformed into *Escherichia coli* (Top10) cells. The cells were cultured on LB plate medium with X-Gal/IPTG resistance screening, and the white bacterial colonies were selected and a cloned library was constructed. The vector primers M13 were used for the sequencing reactions according to the manufacturer’s instructions. The new PCR products were digested by the restriction endonucleases HhaI and RsaI, and the digested products were detected by agarose gel electrophoresis. The DNA fingerprints were analyzed, and the types were designated.

After analyzing the enzyme digestion map, an OTU clone was selected to prepare the puncture tube, which was cultured for 16 h and sent to Shenggong Bioengineering (Shanghai) Co., LTD for sequencing. The sequencing results were inputed into the BLAST program in NCBI to compare with the sequences in the database, and the coverage (C), Shannon diversity index (H′), Simpson index (D) and richness (R) of the clone library were calculated.

#### 2.3.4. Environmental factors in constructed wetland sewage, substrate and air

The wastewater environmental factors of constructed wetland: NH_3_-N, NO_2_^−^-N, NO_3_^−^-N, pH, DO, Water temperature and Redox potential.

Physicochemical indexes of sewage: Ammonia nitrogen (NH_3_-N) was determined by Nessler ‘s reagent spectrophotometry. Nitrite nitrogen (NO_2_^−^-N) was determined by N-(1-naphthyl) ethylenediamine spectrophotometry. Nitrate nitrogen (NO_3_^−^-N) was determined by ultraviolet spectrophotometry. The pH, DO, water temperature and redox potential were measured by a multi-parameter water quality analyzer (HQ30d, HACH, USA).

The soil environmental factors of constructed wetland: pH, Organic matter, Water content, TP, TN, TK.

The physical and chemical indexes of the substrate: The pH was determined by HI2221 pH meter. Organic matter was determined by potassium dichromate volumetric method. The water content was measured by drying at 105°C to constant weight. Total phosphorus (TP) was determined by molybdenum antimony colorimetric method. Total nitrogen (TN) was determined by micro-Kjeldahl method. Total potassium (TK) was determined by flame photometric method.

The air environmental factors of constructed wetland: Temperature (T), Humidity (H), Wind speed (V), O_3_, SO_2_, NO, NO_2_, CO.

The ambient temperature and humidity of the sampling points were measured by TASI-620 digital thermometer and hygrometer. The wind speed was measured by TASI-641 anemometer. Gaseous pollutants O_3_, SO_2_, NO and NO_2_ were measured in real time online by 49C ozone analyzer, 43C sulfur dioxide analyzer and 42C nitrogen oxide analyzer of American thermoelectric company, and CO was monitored in real time by 300 EU CO analyzer.

The above environmental factors were measured using different instruments and methods, as shown in Tables 2–4.

**Table 2:** Physicochemical properties of constructed wetland sewage.

Sampling time	NH_3_-N (mg·L^−1^)	NO_2_^−^-N (mg·L^−1^)	NO_3_^−^-N (mg·L^−1^)	DO (mg·L^−1^)	φ (V)	pH	T (°C)
201804	0.79 ± 0.09	0.11 ± 0.01	18.9 ± 0.33	16.11 ± 1.04	220.1 ± 3.46	7.92 ± 0.62	20.1 ± 1.5
201805	0.66 ± 0.12	0.13 ± 0.03	9.45 ± 0.80	11.45 ± 0.92	238.83 ± 8.25	7.81 ± 0.27	21.6 ± 2.1
201806	0.55 ± 0.04	0.02 ± 0.01	4.6 ± 0.34	6.27 ± 1.06	144.07 ± 9.98	7.66 ± 0.32	27.3 ± 1.4
201807	0.43 ± 0.08	0.06 ± 0.01	5.96 ± 0.44	5.59 ± 0.54	197.93 ± 4.15	7.72 ± 0.19	28.8 ± 1.1
201808	0.47 ± 0.05	0.05 ± 0.01	3.27 ± 0.29	5.06 ± 0.56	62.57 ± 9.54	7.59 ± 0.07	29.8 ± 0.8
201809	0.41 ± 0.03	0.06 ± 0.01	4.11 ± 0.53	4.84 ± 0.95	146.83 ± 3.26	7.75 ± 0.16	24.9 ± 1.9
201810	0.39 ± 0.1	0.02 ± 0.01	9.12 ± 0.94	7.14 ± 1.84	118.03 ± 8.35	7.84 ± 0.24	13.3 ± 0.6
201811	0.72 ± 0.03	0.02 ± 0.01	11.39 ± 1.18	9.11 ± 0.35	185 ± 9.3	7.84 ± 0.24	6.0 ± 1.4
201812	1.22 ± 0.07	0.68 ± 0.06	8.98 ± 0.59	5.86 ± 1.26	145.9 ± 5.01	7.06 ± 0.17	2.6 ± 0.3

**Table 3:** Physical and chemical indexes of soil samples.

Sampling time	pH	Water content(%)	Organic matter (g·kg^−1^)	TN (mg·kg^−1^)	TP (mg·kg^−1^)	TK (mg·kg^−1^)	T (°C)
201804	7.49 ± 0.20	22.24 ± 1.86	15.52 ± 0.85	78.67 ± 3.28	74.93 ± 1.84	71.84 ± 5.73	19.6 ± 1.8
201805	7.85 ± 0.24	29.05 ± 1.48	21.12 ± 0.73	130.34 ± 6.46	112.21 ± 4.17	21.12 ± 1.30	21.6 ± 2.1
201806	6.94 ± 0.99	29.71 ± 1.01	24.25 ± 0.87	109.39 ± 5.11	75.43 ± 4.16	24.25 ± 1.75	26.9 ± 1.0
201807	7.43 ± 0.22	30.1 ± 0.61	21.96 ± 0.78	131.04 ± 2.36	106.02 ± 3.02	21.96 ± 1.47	28.1 ± 0.8
201808	7.61 ± 0.37	27.88 ± 1.55	20.06 ± 1.06	127.08 ± 10.75	126.48 ± 4.91	22.39 ± 1.23	29.1 ± 0.7
201809	7.45 ± 0.29	23.8 ± 0.50	12.58 ± 1.11	67.73 ± 2.81	99.43 ± 5.26	12.58 ± 1.11	24.8 ± 1.5
201810	7.42 ± 0.33	37.05 ± 1.66	36 ± 1.13	154.05 ± 5.86	32.61 ± 1.73	181.48 ± 5.87	13.6 ± 0.5
201811	7.36 ± 0.70	30.4 ± 0.92	39.36 ± 2.34	128.2 ± 6.26	36.45 ± 1.74	157.78 ± 9.94	6.2 ± 1.3
201812	7.59 ± 0.70	25.58 ± 1.31	53.07 ± 3.18	235.1 ± 15.74	34.74 ± 3.35	219.51 ± 7.59	6.1 ± 0.2
201901	8.02 ± 0.30	27.33 ± 1.36	15.45 ± 1.08	108.93 ± 1.25	59.19 ± 3.41	15.45 ± 1.09	3.1 ± 0.9

**Table 4:** Environmental factors of sampling sites.

Sampling time	*T* (°C)	*H* (%)	*V* (m/s)	SO_2_ (μg/m^3^)	NO_2_ (μg/m^3^)	CO (μg/m^3^)	O_3_ (μg/m^3^)	PM_2.5_ (μg/m^3^)	PM_10_ (μg/m^3^)
201804	18.8 ± 1.5	52.1 ± 6.3	2.25 ± 0.85	11.0 ± 0.3	39.3 ± 0.6	0.31 ± 0.02	75.7 ± 1.5	29.1 ± 1.0	98.7 ± 3.8
201805	20.3 ± 2.1	62.4 ± 3.4	1.33 ± 0.80	6.7 ± 0.1	18.3 ± 0.4	0.58 ± 0.04	88.7 ± 0.7	28.3 ± 0.9	41 ± 3.0
201806	26 ± 1.4	84.1 ± 5.1	2.56 ± 0.50	7.0 ± 0.1	19.7 ± 0.5	0.50 ± 0.03	119.7 ± 2.3	26.3 ± 0.4	44.4 ± 1.2
201807	27.5 ± 1.1	94.2 ± 0.9	2.25 ± 0.35	4.0 ± 0.2	8.5 ± 0.5	0.26 ± 0.03	43.5 ± 1.3	8.0 ± 0.2	25.5 ± 0.6
201808	28.5 ± 0.8	86 ± 0.8	2.82 ± 0.18	3.7 ± 0.2	11.0 ± 0.3	0.29 ± 0.03	72.7 ± 1.3	16.0 ± 0.3	30.6 ± 0.9
201809	23.6 ± 1.9	73.5 ± 5.5	2.97 ± 0.27	5.5 ± 0.2	39.5 ± 0.5	0.55 ± 0.02	59 ± 1.1	31.0 ± 1.1	51.0 ± 0.9
201810	12 ± 0.6	63.5 ± 3.4	4.21 ± 0.30	6.1 ± 0.3	26.0 ± 0.3	0.37 ± 0.02	71 ± 0.8	25.0 ± 0.5	52.0 ± 1.1
201811	4.7 ± 1.4	54.3 ± 3.5	6.46 ± 0.60	20.3 ± 1.2	51.3 ± 0.8	1.29 ± 0.03	54.1 ± 0.8	84.3 ± 1.2	139.7 ± 2.8
201812	6 ± 0.3	39.2 ± 2.4	5.22 ± 0.69	20.0 ± 1.3	78.7 ± 0.4	1.41 ± 0.03	25.0 ± 0.8	94.0 ± 1.0	159.7 ± 4.6
201901	4.3 ± 1.5	29.7 ± 2.2	7.74 ± 1.24	18.3 ± 0.2	63.0 ± 1.1	1.60 ± 0.12	29.7 ± 0.6	138.7 ± 0.7	200.0 ± 5.3

#### 2.3.5. The structural differential analysis method of the *Massilia* species group

OriginPro 9.1 was used to draw the analysis map differences of *Massilia* species number and dominance, and the R language tool was used to draw and analyze the Venn diagram of *Massilia’*s population structure correlation.

Redundancy analysis (RDA) and canonical correspondence analysis (CCA) were used to analyze the correlation between environmental factors and the population characteristics of *Massilia* in the corresponding environment. Canoco 5 software was used to analyze the discriminant components of the data. If the length of the first axis was greater than 4.0, CCA should be selected. If between 3.0 and 4.0, RDA and CCA can be selected; if less than 3.0, RDA results are better than CCA. Finally, the RDA or CCA analysis chart is drawn. The contribution rate of environmental factors to the change of population structure was obtained by VPA analysis of R language. The experimental data were analyzed by SPSS 20.0 software.

## 3. Results

### 3.1. Effects of different pairs of primers on the 16S rDNA amplification of *Massilia* spp.

**Figure 3:**
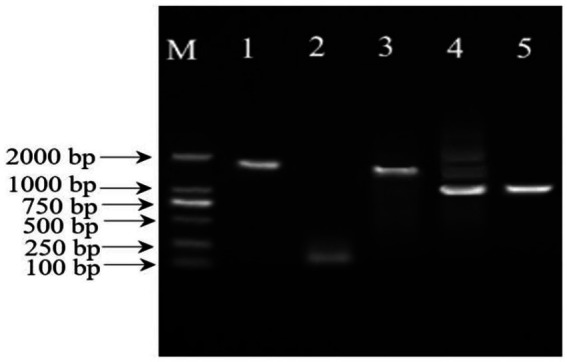
Electrophoresis analysis of PCR products with the five different primers.

The 16S rDNA amplification products of *Massilia* 16S rDNA amplified by different primers were detected by 1% agarose gel electrophoresis (Figure 3 in the Additional files). The result shows that Primers 2, 3 and 4 all had nonspecific amplication production. Primers 1 and 5 had better amplification effects and can be used for 16S rDNA clone library construction.

### 3.2. Analysis of the enzyme digestion products for the amplification of two pairs of primers

**Figure 4:**
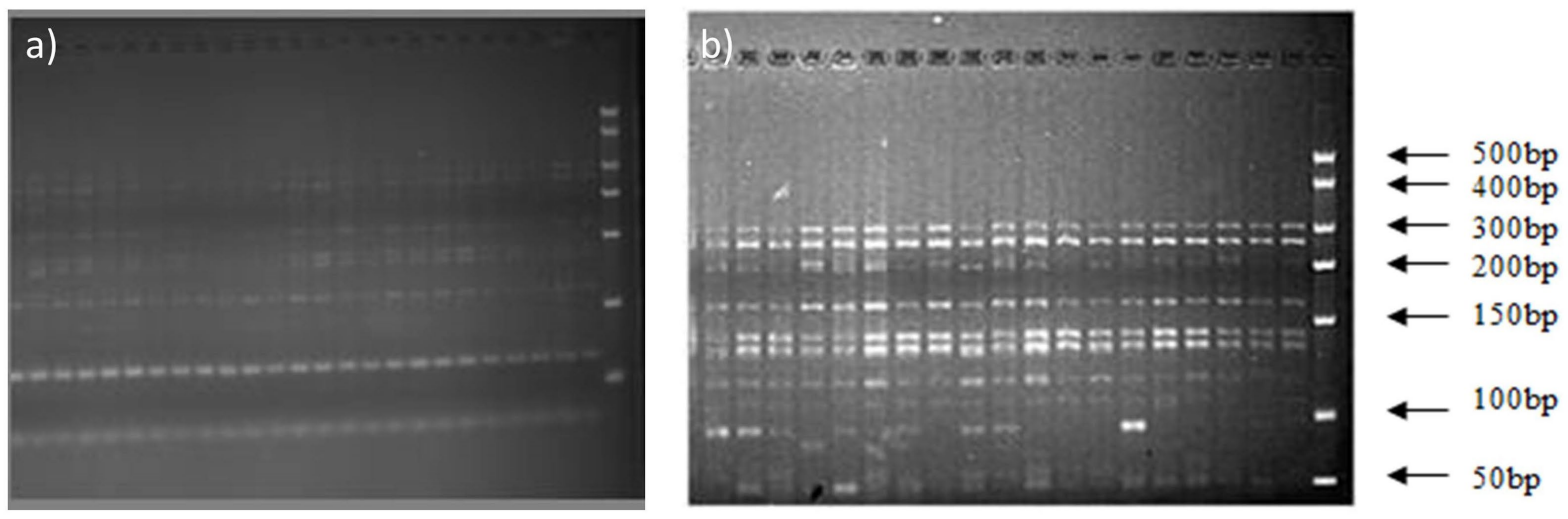
Electrophoresis analysis of amplified 16S rDNA digested by HhaI and RsaI. **(A)** Electrophoretic Strips of 16S rDNA Fragments Amplified by Primer 1; **(B)** Electrophoretic Strips of 16S rDNA Fragments Amplified by Primer 5.

The 16S rDNA of *Massilia* spp. was amplified by using primers No. 5 and No. 1. Then, the amplified products were digested with the endonucleases RsaI and HhaI, and the digested products were detected by 3% agarose gel electrophoresis. The results shows that the digested products of 16S rDNA in the clone library amplified by primer No. 1 had similar fragments (Figure 4A in the Additional files), while the digested products of 16S rDNA amplified by primer No. 5 had discrepancies (Figure 4B).

**Figure 5:**
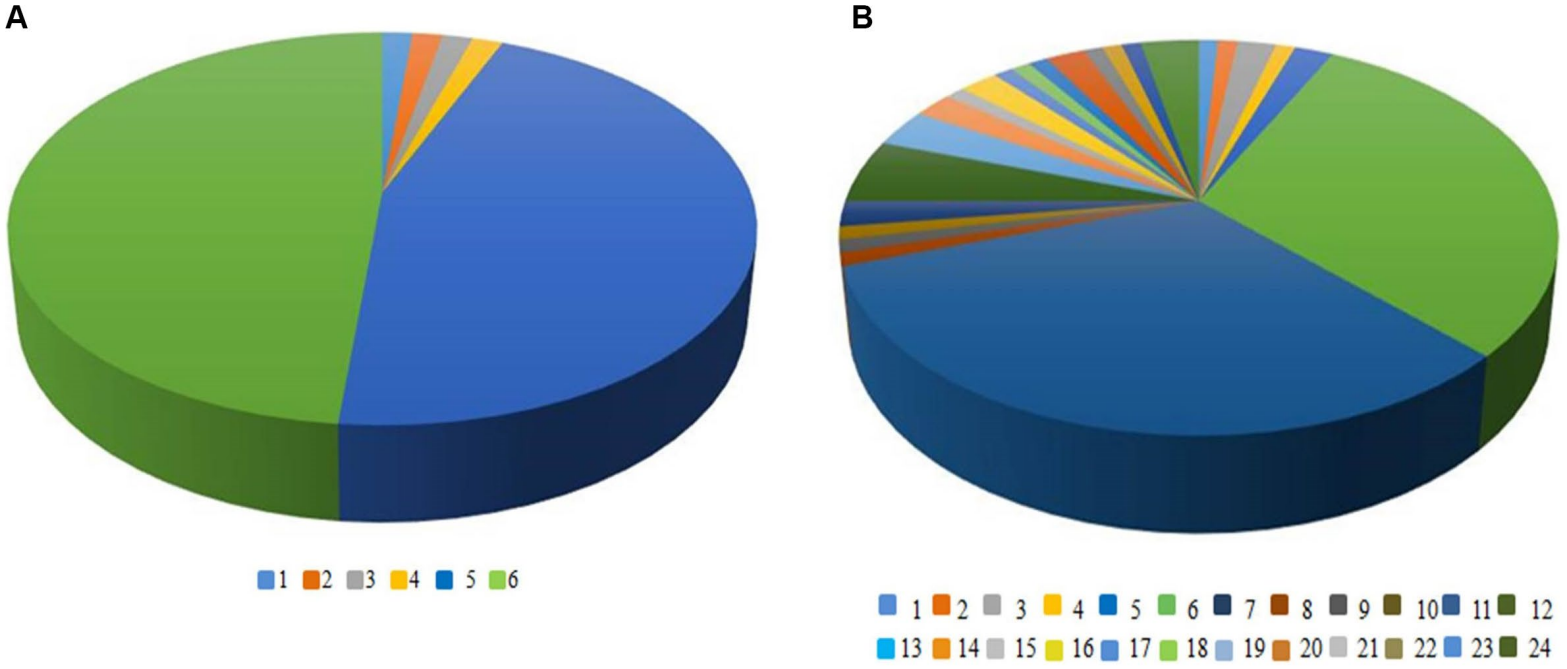
OTUs in the constructed 16S rDNA clone libraries amplified by different pairs of primers. **(A)** Amplification of OTU in 16S rDNA clone library with primer 1; **(B)** Amplification of OTU in 16S rDNA clone library with primer 5.

Figure 5 shows the OTUs in the constructed 16S rDNA clone libraries amplified by primers No. 1 and No. 5. There were 6 OTUs in the clone library amplified by primer No. 1, while there were 24 OTUs in the clone library amplified by primer No. 5. This indicated that the 16S rDNA amplified products of primer No. 5 were better in distinguishing the diversity of *Massilia* spp. Therefore, primer No. 5 was chosen for 16S rDNA clone library construction of *Massilia* spp.

### 3.3. Diversity analysis of the *Massilia* spp. in constructed wetlands

The specific alpha diversity indices are shown in Table 5. Through the coverage index, the coverage of the 16S rDNA clone libraries of *Massilia* spp. in the constructed wetland was greater than 86%, which indicated that most of the *Massilia* spp. were included in the constructed clone libraries. Large differences in the richness of *Massilia* spp. could be observed every month. The sampling in summer had the highest abundance, while the lowest abundance was in the samples collected in the winter. The richness of *Massilia* spp. was obviously different in the constructed wetland in different seasons and was higher in the summer and autumn than in the spring and winter. The Shannon index and Simpson index were selected to estimate the diversity of *Massilia* spp. According to the Shannon index and Simpson index, the samples in the summer had the highest community diversity.

**Table 5:** The diversity index of *Massilia* based on bacteria clone libraries in different environment of constructed wetland.

Number	Sample name	Coverage(%)	Richness(R)	Shannon(H′)	Simpson(D)
1	AprA	91.38	6.67	3.28	0.9
2	AprL	86.85	8.22	3.52	0.89
3	AprW	95.55	8.16	3.36	0.94
4	AprS	89.77	7.48	2.97	0.87
5	AprR	90.74	7.35	3.15	0.86
6	MayA	93.02	8.04	3.64	0.91
7	MayL	94.1	7.68	3.85	0.93
8	MayW	94.07	7.15	3.91	0.92
9	MayS	95.27	9.23	3.83	0.89
10	MayR	94.23	8.64	3.77	0.91
11	JunA	94.69	7.89	3.56	0.94
12	JunL	93.75	8.74	3.28	0.89
13	JunW	95.16	8.16	3.36	0.92
14	JunS	92.42	7.66	3.47	0.9
15	JunR	93.79	7.98	3.69	0.94
16	JulA	93.5	9.32	3.84	0.88
17	JulL	92.91	10.49	3.91	0.86
18	JulW	90.12	12.56	4.35	0.95
19	JulS	97.41	13.11	5.54	1
20	JulR	93.2	13.08	5.01	0.96
21	AugA	91.03	10.27	4.98	1
22	AugL	91.41	12.32	5.32	0.97
23	AugW	91.28	11.09	5.47	0.99
24	AugS	93.38	12.46	5.66	0.99
25	AugR	99.22	11.45	5.73	1
26	SepA	88.59	12.74	5.97	0.98
27	SepL	92.5	12.12	5.82	0.97
28	SepW	96.98	12.88	5.43	1
29	SepS	96.73	12.65	4.9	0.99
30	SepR	95.92	12.37	4.58	1
31	OctA	96.69	9.97	3.92	0.91
32	OctL	95.81	9.78	3.58	0.89
33	OctW	96.4	9.61	3.74	0.92
34	OctS	93.63	9.54	3.65	0.91
35	OctR	91.24	8.76	3.94	0.93
36	NovA	97.22	7.54	3.22	0.86
37	NovL	91.18	8.55	3.14	0.9
38	NovW	92.68	7.31	3.26	0.91
39	NovS	95.12	6.98	3.43	0.89
40	NovR	97.31	7.85	3.59	0.92
41	DecA	96.87	8.52	3.62	0.86
42	DecW	97.23	7.68	3.57	0.87
43	DecS	94.25	7.94	3.63	0.89
44	DecR	95.49	8.26	3.59	0.91
45	JanA	93.68	8.23	3.31	0.9
46	JanS	93.86	9.57	3.53	0.89
47	JanR	91.82	8.85	3.25	0.88

### 3.4. The community structure of *Massilia* spp. in a constructed wetland sewage treatment system

#### 3.4.1. The community structure of *Massilia* spp. in sewage of constructed wetland

**Figure 6:**
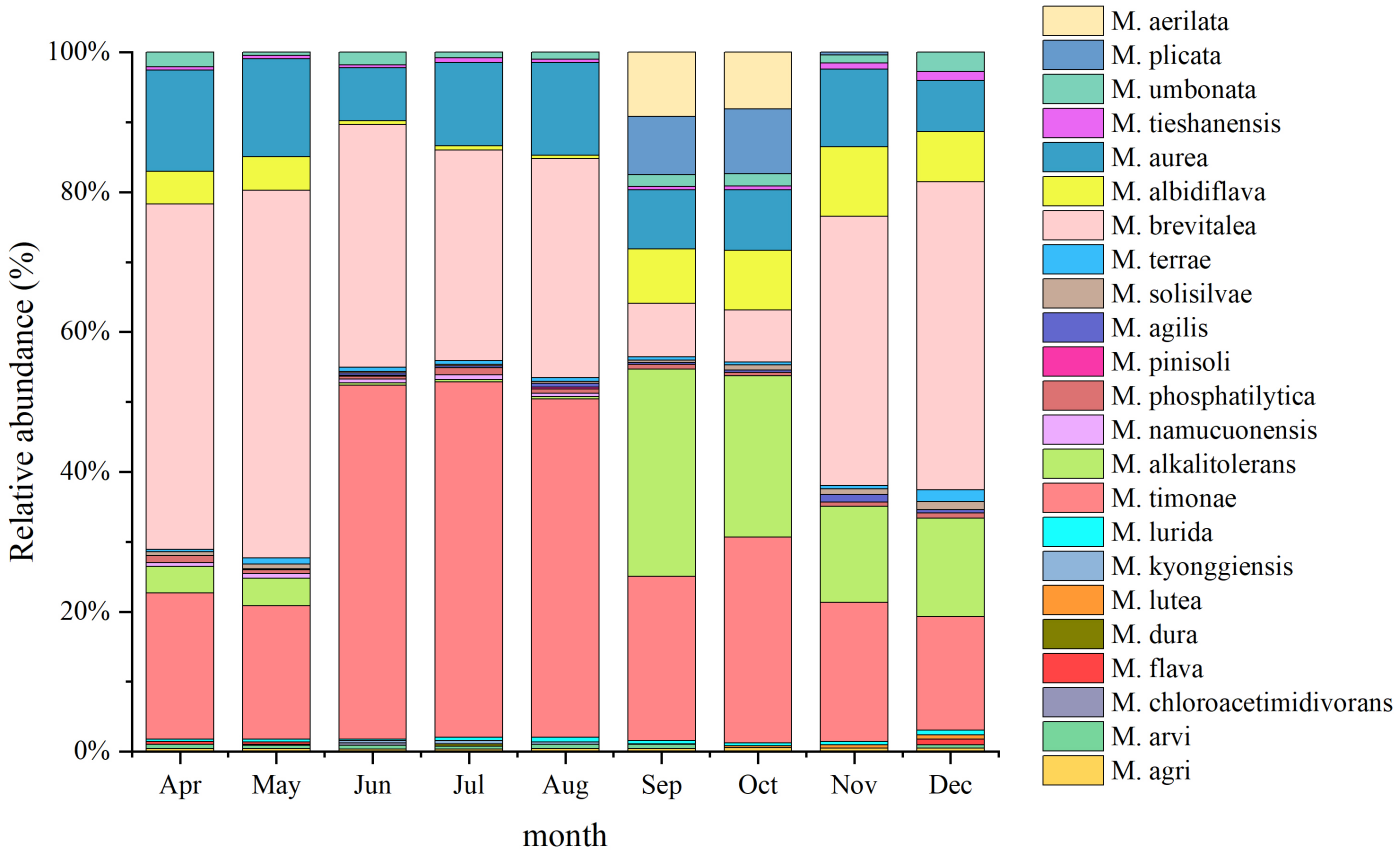
Analysis of the 16S rDNA clone library based on microbes in sewage.

A total of 23 *Massilia* species were found in constructed wetland sewage (Figure 6). There were significant differences in the number of dominant species of *Massilia* spp. in different months. There was a significant temporal variation in the distribution of dominant *Massilia* spp. The relative abundance of *M. albidiflava* was the highest in November. From June to August, the relative abundance was less than 1%, which was different from that in other seasons. The relative abundance of *M. alkalitolerans* was the highest in September, which was no more than 0.5% relative abundance from June to August. The relative abundance of *M. aurea* was more than 7% over the year, and there was no significant difference in different months. The relative abundance of *M. brevitalea* was highest in the summer and lowest in the autumn, which was similar to the variation in *M. brevitalea* in the substrate and rhizosphere. The relative abundance of *M. timonae* was highest in the summer, with approximately 20% relative abundance in other seasons. The relative abundance of *M. umbonata* was lowest in May and highest in December, and the seasonal variation was insignificant.

**Figure 7:**
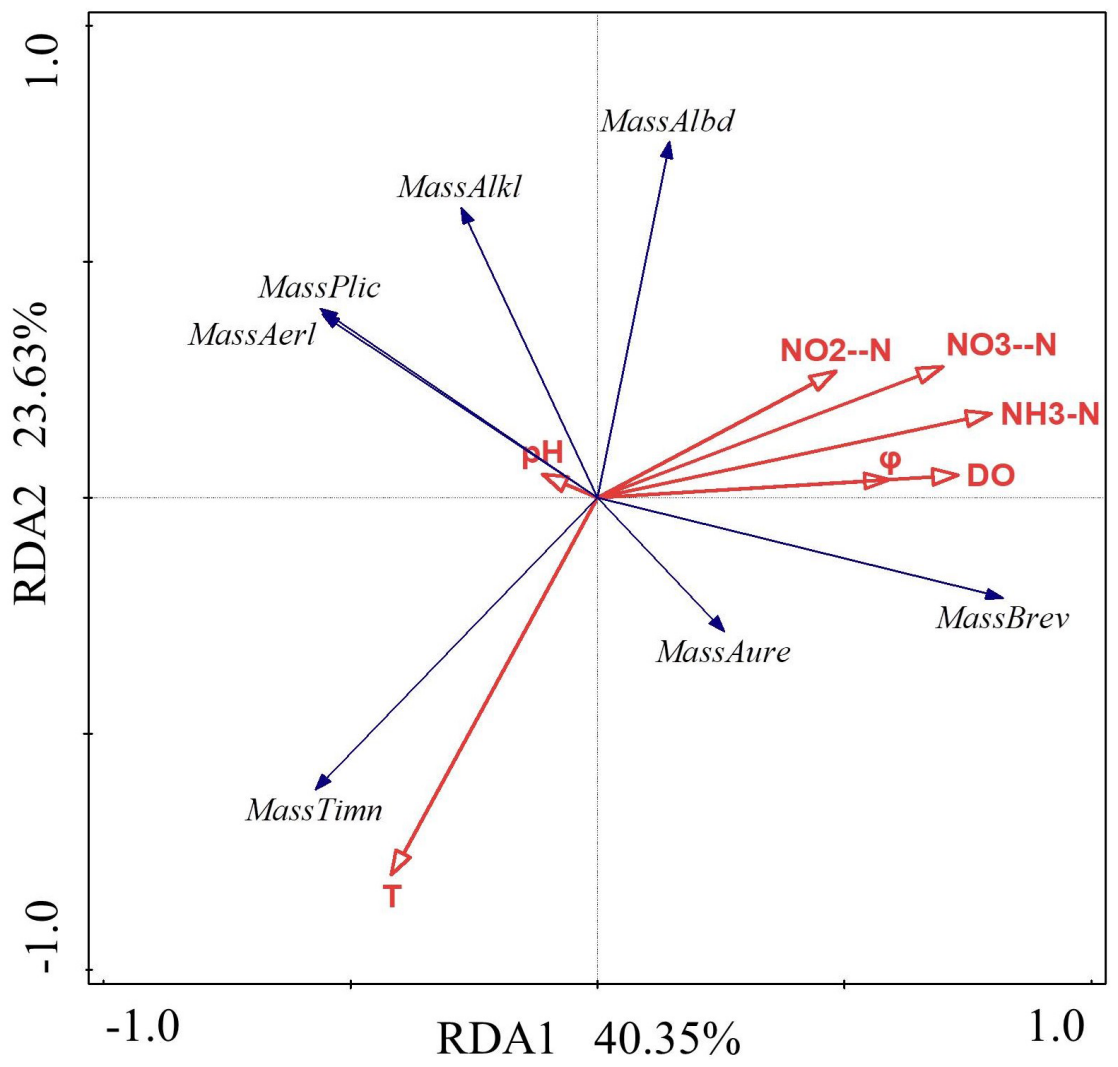
Redundancy analysis of *Massilia* and environmental factors in wastewater.

The data were analyzed with Canoco 5 software. The findings indicated that the RDA linear model should be chosen for analysis because the length of the first axis was less than 3.0. The findings reveal that the first axis’ interpretation rate is 40.35%, the second axis’ interpretation rate is 23.63%, and the combined interpretation rate of the two axes is 63.9%. It demonstrates that the structure of *Massilia* species is significantly influenced by environmental factors in constructed wetland sewage. According to Figure 7 and Table 6, the dominant species *M. timonae* was highly significantly positively correlated with T (*p* < 0.01), and highly significantly negatively correlated with NH_3_-N and NO_3_^−^-N (*p* < 0.01). *M. brevitalea* was highly significantly positively correlated with NH_3_-N, NO_3_^−^-N, DO and φ (*p* < 0.01). *M. albiflava* was highly significantly negatively correlated with T (*p* < 0.01). *M. aurea* was significantly positively correlated with φ (*p* < 0.05). *M. plicata* and *M. aerilata* were significantly negatively correlated with NH_3_-N and φ (*p* < 0.05). The results of the VPA analysis showed that the four factors with the highest contribution rates were NH_3_-N, T, DO and NO_3_^−^-N, which were 26.5, 22.2, 21.8 and 21.8%. The findings revealed that the aforementioned four factors had the greatest influence on the composition and distribution of the *Massilia* community in constructed wetland sewage.

**Table 6:** Correlation analysis of dominant species of *Massilia* and environmental factors in sewage.

	NH_3_-N	DO	NO_3_^−^-N	NO_2_^−^-N	φ	pH	*T*
*M. timonae*	−0.577**	−0.324	−0.541**	−0.183	−0.248	0.078	0.728**
*M. alkalitolerans*	−0.029	0.114	0.273	0.052	−0.149	0.210	−0.378
*M. brevitalea*	0.579**	0.537**	0.511**	0.118	0.629**	−0.060	−0.174
*M. albidiflava*	0.112	0.194	0.371	−0.061	−0.044	0.243	−0.725**
*M. aurea*	0.209	0.287	0.280	−0.006	0.392*	0.291	0.249
*M. plicata*	−0.423*	−0.216	−0.124	−0.058	−0.427*	0.201	−0.050
*M. aerilata*	−0.433*	−0.218	−0.127	−0.077	−0.419*	0.195	−0.027

**Significant correlation at 0.01 level (bilateral); *Significant correlation at the 0.05 level (bilateral).

#### 3.4.2. The community structure of *Massilia* spp. in constructed wetland substrate

**Figure 8:**
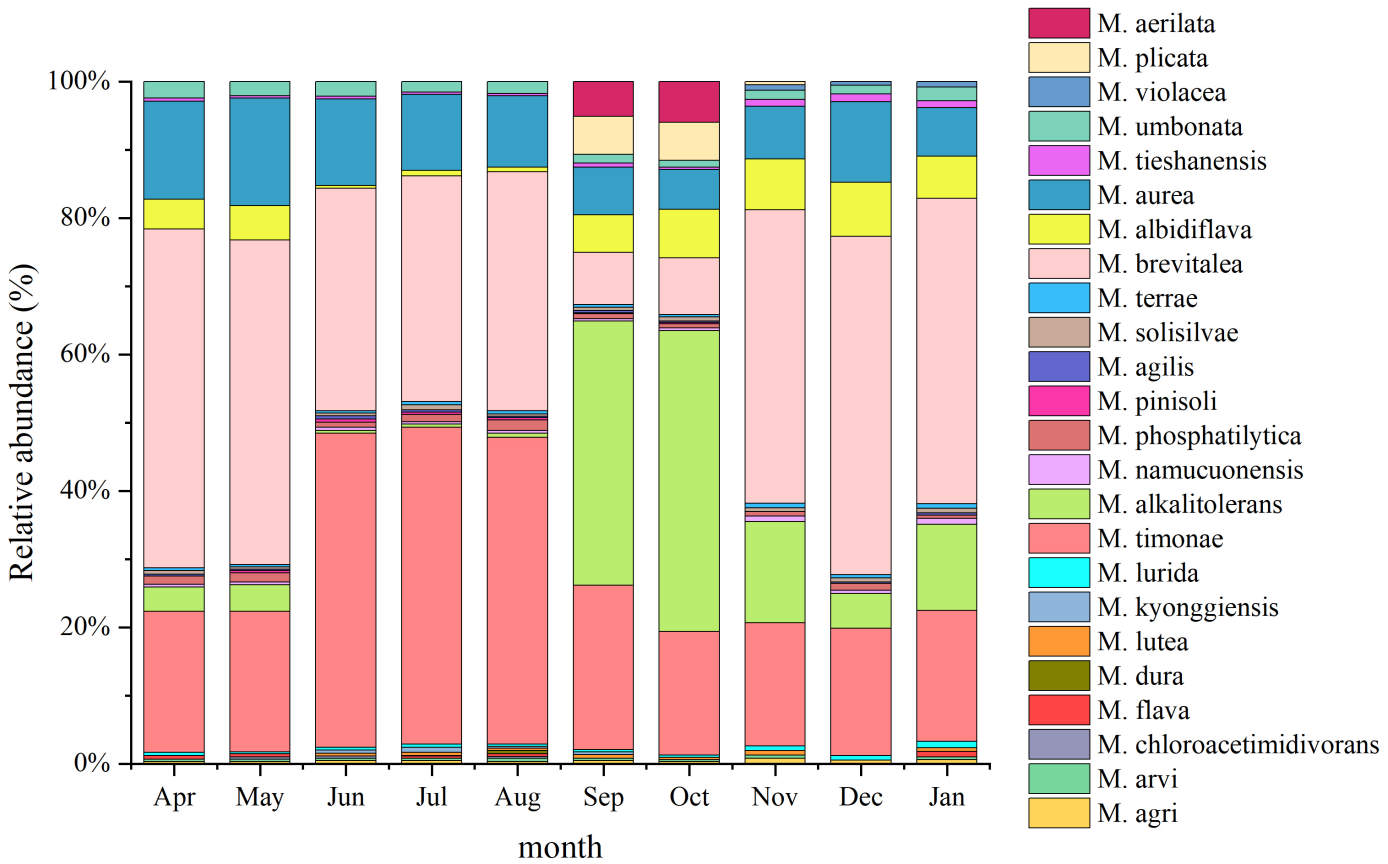
Analysis of the 16S rDNA clone library based on soil microbes.

A total of 24 *Massilia* species were found in the constructed wetland substrate (Figure 8). The dominant species in the constructed wetland substrate were *M. albidiflava*, *M. alkalitolerans*, *M. aurea*, *M. brevitalea*, *M. timonae* and *M. umbonata.* The relative abundance of *M. albidiflava* was the lowest in the summer, which was significantly different from other seasons. There was no significant difference in the relative abundance of *M. albidiflava* in the other seasons. The relative abundance of *M. alkalitolerans* in the substrate decreased in order from autumn to winter to spring to summer. The relative abundance of *M. aurea* was higher in the spring and summer than in the autumn. The relative abundance of *M. brevitalea* was highest in the spring and winter and lowest in the autumn, and there were obvious seasonal differences. The relative abundance of *M. timonae* in the summer was significantly higher than that in the other three seasons. There was no significant difference in the relative abundance of *M. timonae* between months in the same season. The relative abundance of *M. umbonata* was lower all year.

**Figure 9:**
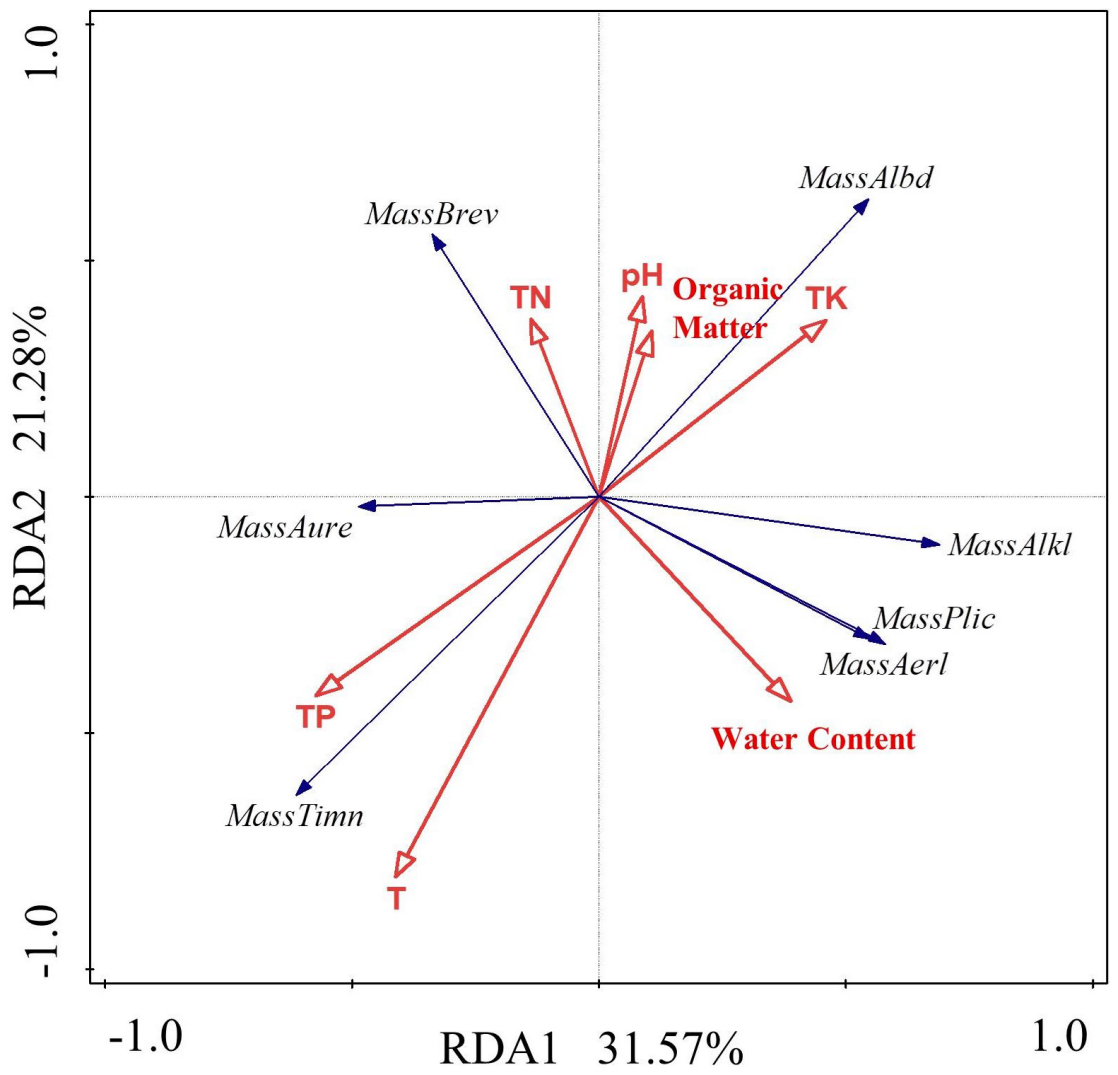
Redundancy analysis of *Massilia* and environmental factors in soil.

The RDA linear model was used to analyze the data. The results show that the interpretation rate of the first axis is 31.57%, the second axis is 21.18%, and the common interpretation rate of the two axes is 52.75%. It shows that environmental factors in the constructed wetland substrate have a significant effect on the structure of *Massilia* spp. According to Figure 9 and Table 7, the dominant species *M. timonae* was very significantly positively correlated with T and TP content (*p* < 0.01). *M. alkalitolerans* was significantly negatively correlated with TP (*p* < 0.05). *M. brevitalea* was highly significantly positively correlated with TN (*p* < 0.01). *M. albiflava* was very significantly positively correlated with TN (*p* < 0.01), and highly significantly negatively correlated with TP and T (*p* < 0.01). *M. aurea* was significantly positively correlated with TP (*p* < 0.05). *M. plicata* and *M. aerilata* were positively correlated with water content and T. The results of the VPA analysis showed that the three factors with the highest contribution rates were T, TP and TN, which were 19.1, 14.1 and 9.7%. The findings revealed that the aforementioned three factors had the greatest influence on the composition and distribution of the *Massilia* community in constructed wetland substrate.

**Table 7:** Correlation analysis of dominant species of *Massilia* and environmental factors in soil.

	pH	TN	Water content	TK	TP	Organic matter	*T*
*M. timonae*	−0.346	−0.027	−0.161	−0.360	0.570**	−0.132	0.749**
*M. alkalitolerans*	0.145	−0.127	0.191	0.108	−0.461*	0.004	−0.208
*M. brevitalea*	0.205	0.840**	−0.173	0.269	−0.138	0.059	−0.315
*M. albidiflava*	0.137	0.534**	0.124	0.318	−0.613**	0.112	−0.780**
*M. aurea*	0.053	−0.026	−0.243	0.054	0.362*	−0.054	0.344
*M. plicata*	−0.088	−0.140	0.130	−0.040	−0.164	−0.030	0.062
*M. aerilata*	−0.109	−0.134	0.132	−0.040	−0.158	−0.032	0.044

**Significant correlation at 0.01 level (bilateral); *Significant correlation at the 0.05 level (bilateral).

#### 3.4.3. The community structure of *Massilia* spp. in the rhizosphere sample of constructed wetland plants

**Figure 10:**
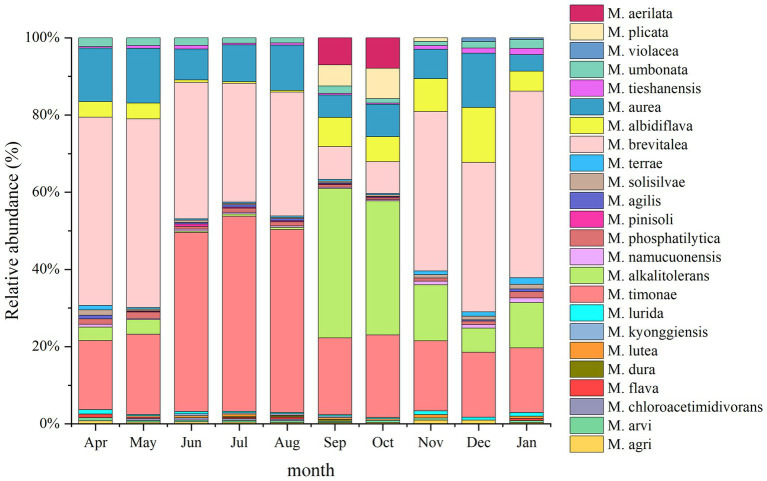
Analysis of the 16S rDNA clone library based on rhizosphere microbes.

A total of 24 *Massilia* species were found in the rhizosphere of constructed wetland plants (Figure 10). The dominant species in the rhizosphere sample of constructed wetland plants were *M. albidiflava*, *M. alkalitolerans*, *M. aurea*, *M. brevitalea*, *M. timonae*, and *M. umbonata*. The relative abundance of *M. albidiflava* was higher in the autumn and winter than in the spring and summer. The relative abundance of *M. albidiflava* was lowest in July and highest in December. The variation in the relative abundance of *M. albidiflava* in the rhizosphere sample of plants was similar to that in the substrate sample of the constructed wetland. The relative abundance of *M. alkalitolerans* was higher in the autumn and winter than in the spring and summer, and the relative abundance was lowest in the summer (less than 1%). The variation in relative abundance for *M. aurea* was relatively slight. The relative abundance of *M. aurea* was higher in spring and summer than in autumn and winter, and the relative abundance was lowest in January and highest in May. The relative abundance of *M. brevitalea* was higher in the spring and summer than in the autumn and winter, and the relative abundance was highest in the summer, at nearly 50%. In the autumn, the relative abundance of *M. brevitalea* was lower in September and October, at approximately 8%. The relative abundance of *M. timonae* was the highest from June to August, up to approximately 47%. The relative abundance in the other months was approximately 20%. The variation in the relative abundance of *M. timonae* in the rhizosphere sample of plants was similar to that in the substrate sample of constructed wetland. The relative abundance of *M. umbonata* was lower than 2.5% all year, and was higher in the spring and winter than in the summer and autumn.

#### 3.4.4. The community structure of *Massilia* spp. in the phyllosphere sample of constructed wetland plants

**Figure 11:**
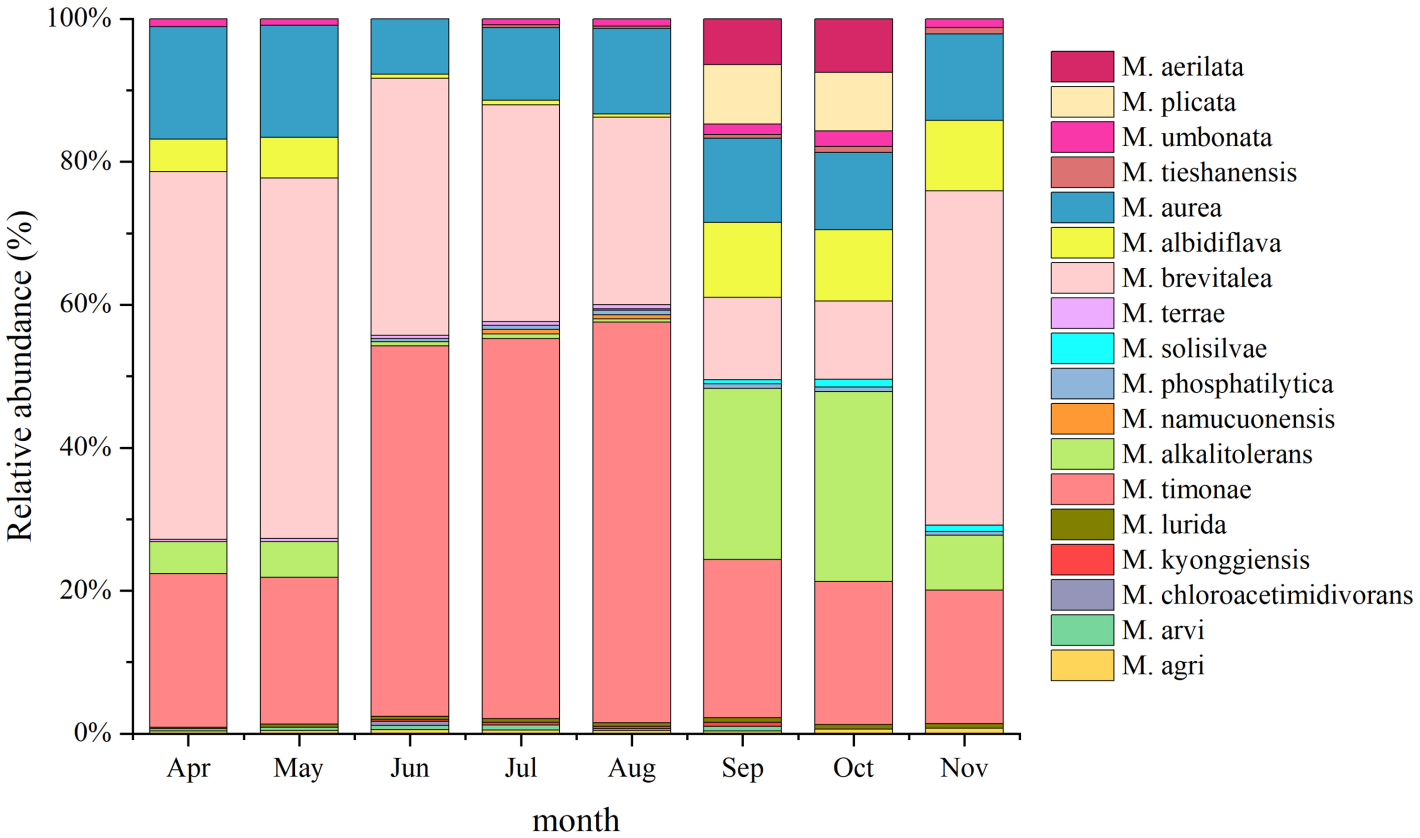
Analysis of the 16S rDNA clone library based on phyllosphere microbes.

A total of 18 *Massilia* species were found in the phyllosphere sample of constructed wetland plants (Figure 11). The relative abundance of *M. albidiflava* in the phyllosphere sample decreased in an order from autumn to spring to summer. The relative abundance of *M. albidiflava* was highest in the autumn and lowest in the summer. The variation regularity of the relative abundances of *M. alkalitolerans* was similar to that of *M. albidiflava* in the phyllosphere sample. There was no obvious seasonal variation in the relative abundance of *M. aurea*. The relative abundance of *M. brevitalea* exhibited significant differences between seasons, with the highest relative abundance observed in the spring and the lowest relative abundance observed in autumn. There were obvious seasonal differences in the relative abundance of *M. timonae*; the highest relative abundance was approximately 50% in the summer, and the lowest relative abundance was approximately 19% in the spring and autumn. The relative abundance of *M. umbonata* was similar to that in the other environments of constructed wetlands, and its relative abundance was lower.

#### 3.4.5. The community structure of *Massilia* spp. in the air sample of constructed wetland plants

**Figure 12:**
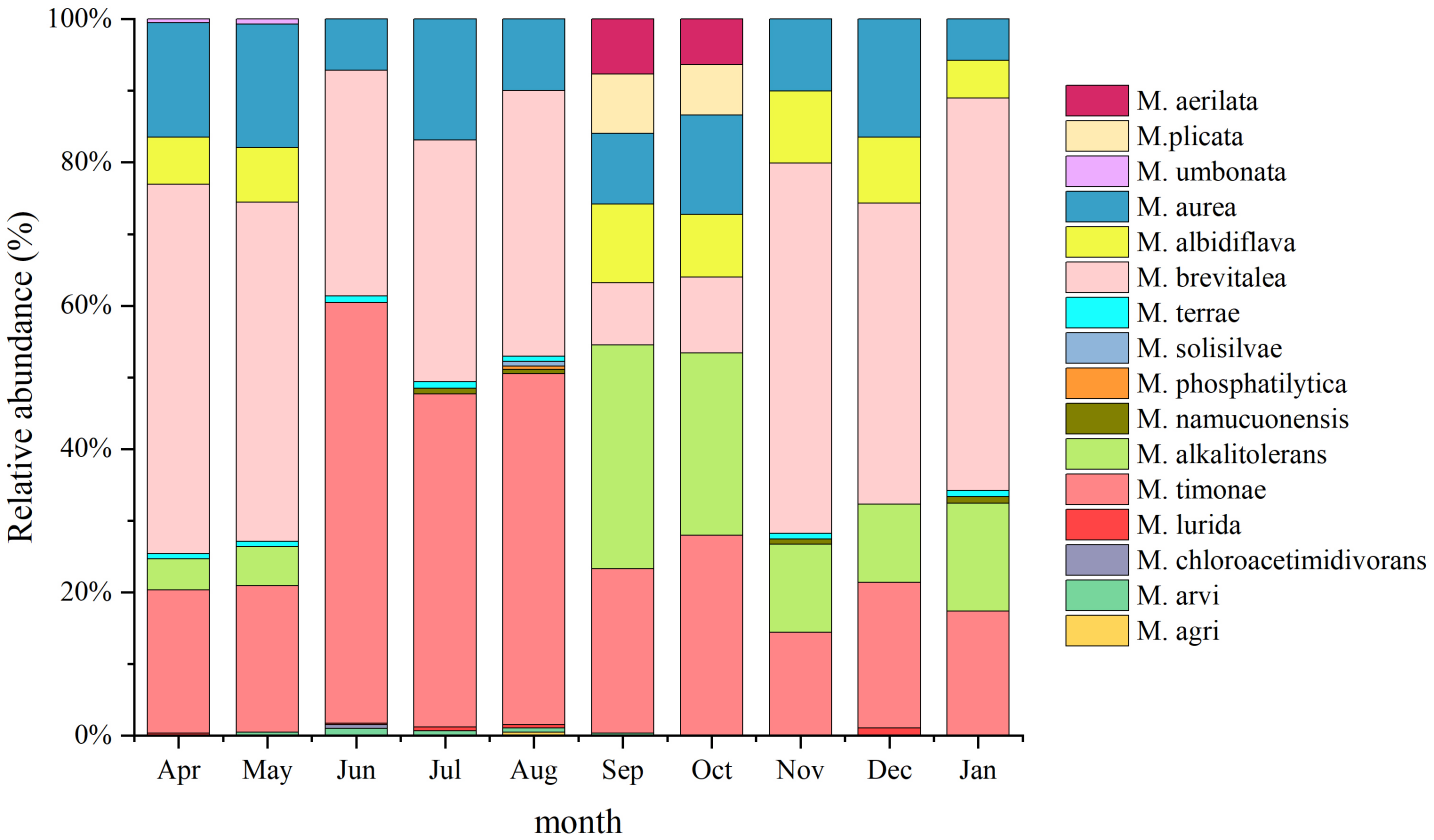
Analysis of the 16S rDNA clone library based on airborne microbes.

A total of 16 *Massilia* species were found in the air sample of the constructed wetland (Figure 12). The relative abundances of *M. albidiflava* and *M. alkalitolerans* exhibited significant seasonal variation; the relative abundance was higher in the autumn and winter, while the species were not detected in the summer. The variation in the relative abundance of *M. aurea* between seasons was not obvious; the relative abundance was highest in April, May, July and December (approximately 16%), while it was lowest in January. The relative abundance of *M. brevitalea* was similar to that in other environments of constructed wetlands, with the highest relative abundance observed in January and the lowest relative abundance observed in September and October. The relative abundance of *M. timonae* exhibited obvious seasonal variation, with the highest relative abundance observed in the summer, while there were no significant differences among the spring, autumn and winter.

**Figure 13:**
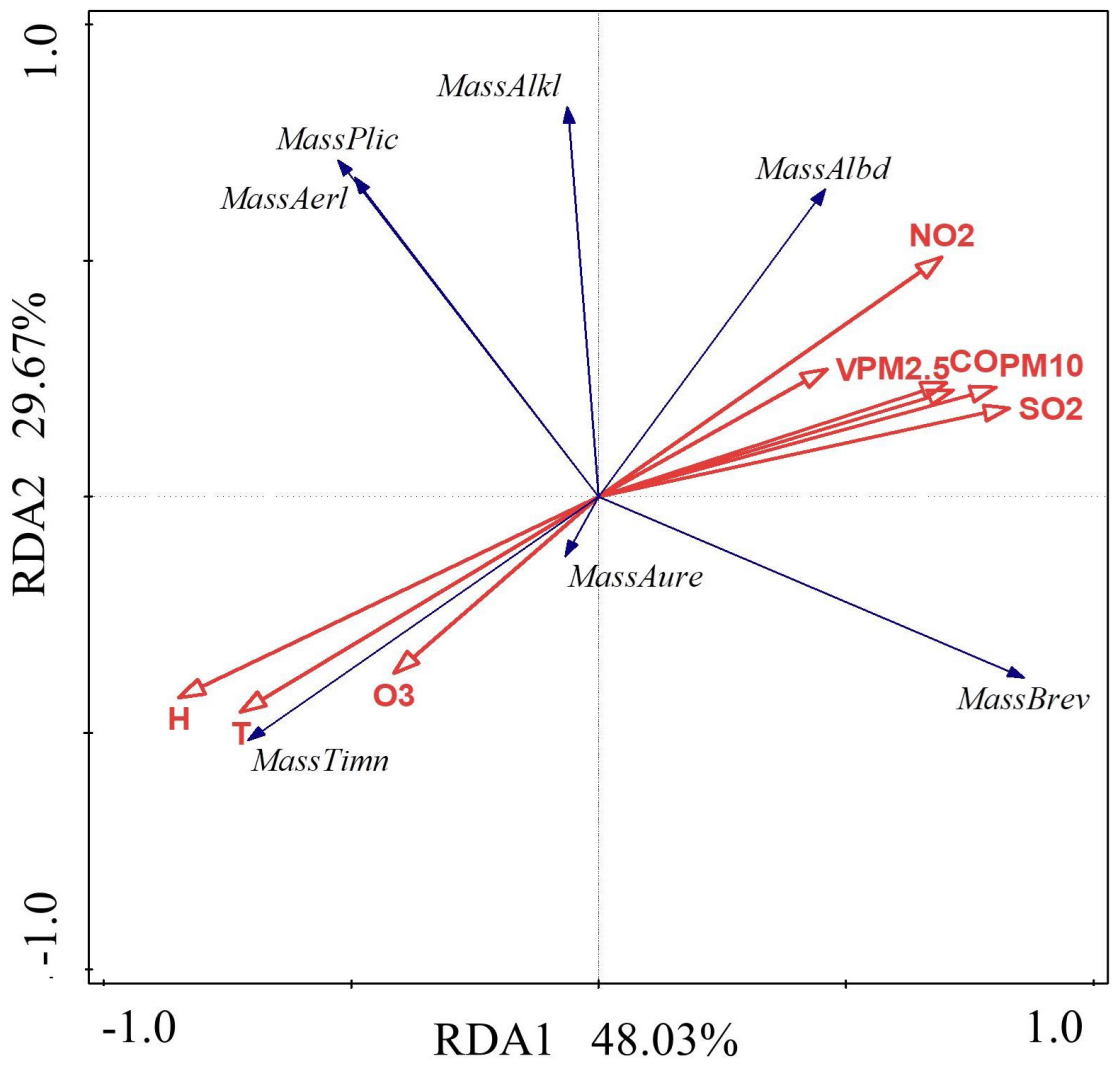
Redundancy analysis of *Massilia* and environmental factors in air.

The data in Table 4 and Figure 12 were examined using the RDA model. The results show that the first axis interpretation rate is 48.03%, the second axis interpretation rate is 29.67%, and the combined interpretation rate of the two axes is 77.69%. It was discovered that the air environmental factors of the constructed wetland had a substantial impact on the community structure of *Massilia*. As shown in Figure 13 and Table 8, the dominant species of the genus *M. timonae* in the air of constructed wetlands was highly significantly positively correlated with temperature, humidity and O_3_ (*p* < 0.01), highly significantly negatively correlated with SO_2_, NO_2_, CO, PM_2.5_ and PM_10_ (*p* < 0.01), and significantly negatively correlated with wind speed (*p* < 0.05); *M. alkalitolerans* showed a highly significant positive correlation with wind speed (*p* < 0.01), a significant positive correlation with NO_2_ (*p* < 0.05), and a highly significant negative correlation with humidity (*p* < 0.01); *M. albidiflava* showed highly significant positive correlations (*p* < 0.01) with SO_2_, NO_2_ and PM_10_, significant positive correlations (*p* < 0.05) with PM_2.5_ and CO, and highly significant strong negative correlations (*p* < 0.01) with temperature and humidity; *M. aurea* showed highly significant negative correlations (*p* < 0.01) with wind speed and PM_2.5_ and significant negative correlations (*p* < 0.05) with CO and PM_10_. The results obtained from the VPA analysis showed that humidity, NO_2_, temperature and SO_2_ were the four factors that explained the greatest contribution with values of 40.1, 11.4, 15.2 and 5.1%. The findings revealed that the community composition and distribution of *Massilia* in the air of the constructed wetland is most influenced by the above four factors.

**Table 8:** Correlation analysis of dominant species of *Massilia* and environmental factors in the air.

	*T*	*H*	*V*	SO_2_	NO_2_	CO	O_3_	PM_2.5_	PM_10_
*M. timonae*	0.731**	0.778**	−0.377*	−0.664**	−0.729**	−0.588**	0.475**	−0.587**	−0.658**
*M. alkalitolerans*	−0.36	−0.503**	0.500**	0.15	0.430*	0.233	−0.324	0.225	0.208
*M. brevitalea*	−0.463**	−0.683**	0.276	0.659**	0.420*	0.539**	−0.222	0.562**	0.628**
*M. albidiflava*	−0.607**	−0.568**	0.262	0.503**	0.641**	0.457*	−0.357	0.428*	0.474**
*M. aurea*	0.338	0.297	−0.702**	−0.318	−0.339	−0.443*	0.18	−0.507**	−0.430*
*M. plicata*	0.022	0.144	0.109	−0.343	−0.068	−0.262	0.026	−0.247	−0.275
*M. aerilata*	0.016	0.147	0.093	−0.323	−0.062	−0.244	0.024	−0.234	−0.261

**Significant correlation at 0.01 level (bilateral); *Significant correlation at the 0.05 level (bilateral).

### 3.5. The influence of environmental factors on the structure of the *Massilia* spp. community in constructed wetlands

**Figure 14:**
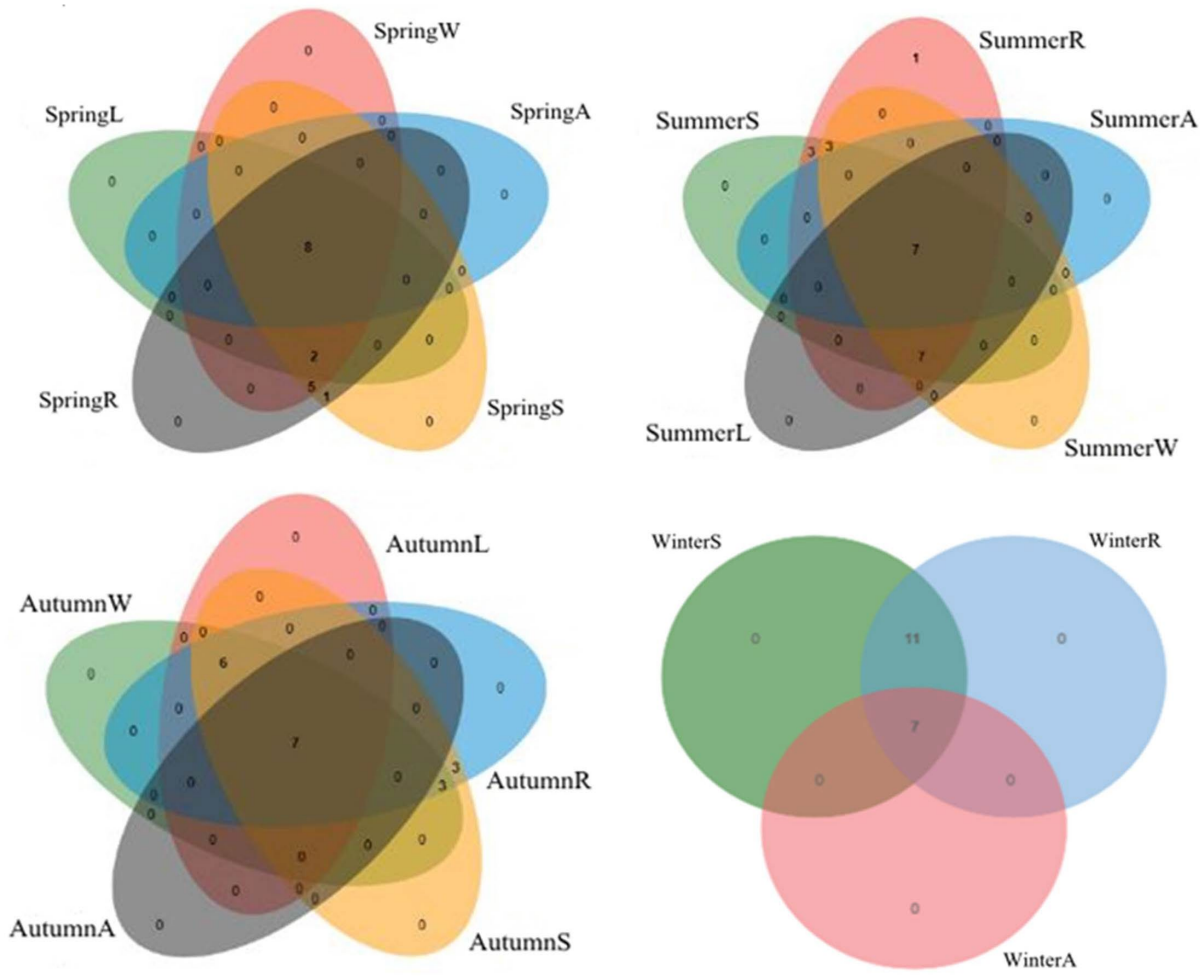
Correlation of *Massilia* spp. in four seasons. (A): *Massilia* in the Air; (L): *Massilia* in the Leaf (Phyllosphere); (W): *Massilia* in the Water (Sewage); (S): *Massilia* in the Substrate (Soil); (R): *Massilia* in the Rhizosphere.

The structure of *Massilia* spp. in different seasons and different environments was analyzed by a Venn diagram (Figure 14). The species numbers of *Massilia* spp. in the constructed wetland sewage, substrate, plant rhizosphere, plant phyllosphere and air samples were 15, 16, 16, 10 and 8 species, respectively in the spring, 17, 20, 21, 14 and 7 species, respectively in the summer, and 13, 19, 19, 13 and 7 species, respectively in the autumn. The species number of *Massilia* spp. in the substrate and rhizosphere in the winter was 18, while it was 7 in the air. The above results show that the species of *Massilia* in the air samples were all found in the other environments of the constructed wetland. Therefore, it can be concluded that *Massilia* spp. in the air were probably from the underlying surface of the constructed wetland.

**Figure 15:**
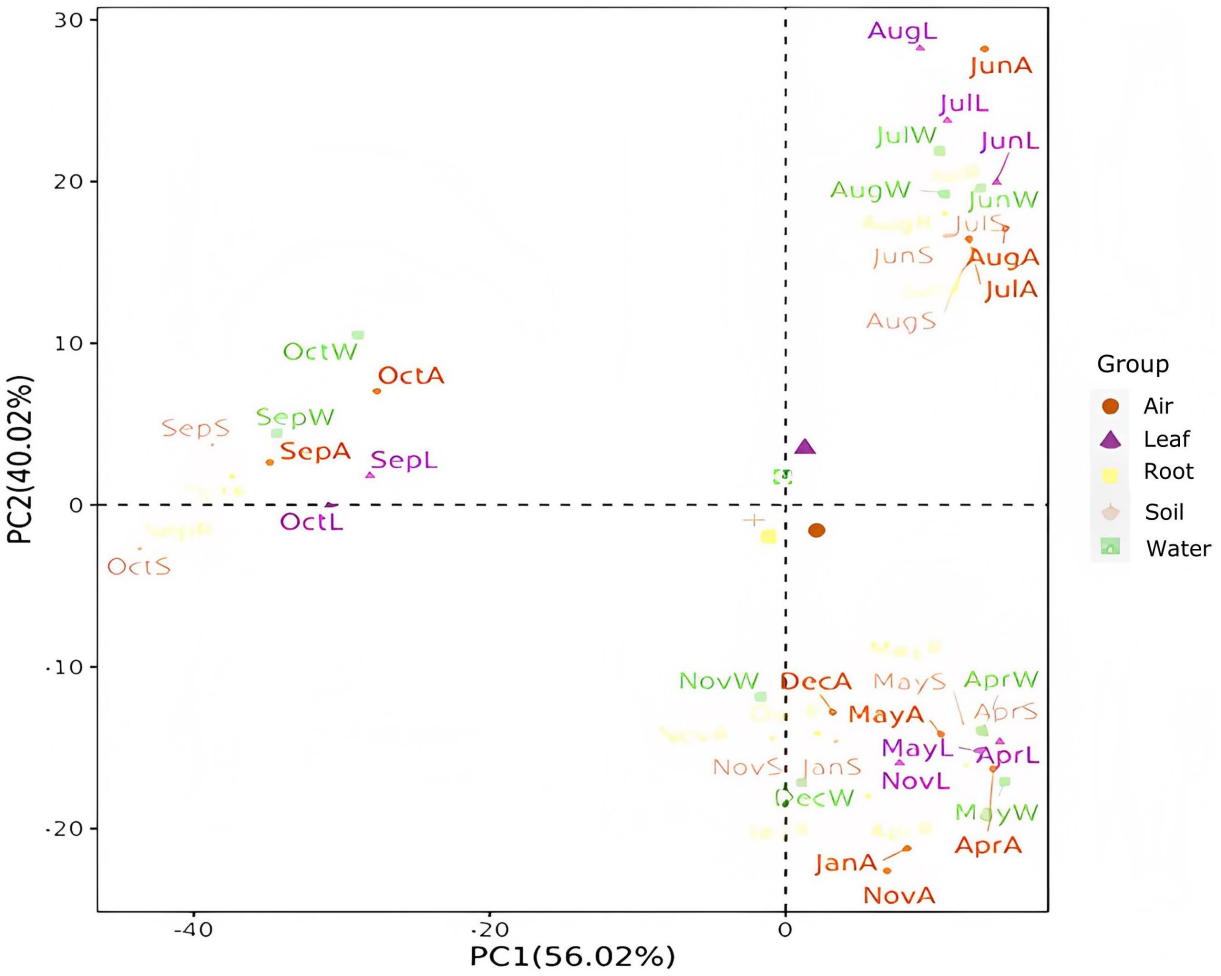
PCA of the *Massilia* population composition in different months and environments. (Air): *Massilia* in the Air; (Leaf): *Massilia* in the Phyllosphere; (Root): *Massilia* in the Rhizosphere; (Soil): *Massilia* in the Substrate; (Water): *Massilia* in the Sewage.

PCA was employed to analyze the composition of *Massilia* species in each environmental sample of the constructed wetland, and the distribution characteristics among the samples were described by two-dimensional images, as shown in Figure 15. The results showed that there were significant differences in the composition of *Massilia* spp. among different environmental samples, while the differences in different seasons were small. Most interestingly, the study found that the similarity of *Massilia* spp. in different environmental samples within the same season was higher than that in the same environmental sample within different seasons.

## 4. Discussion

Since *Massilia* was first discovered by La Scola in 1998, scholars from all over the world have successfully discovered other *Massilia* from different regions and environments and studied them. In 2003, Wery et al. studied the microbial community in Antarctic soil, isolated a strain capable of producing protease, used universal primers to amplify 16S rDNA by PCR, and determined the phylogenetic relationship of the strain by gene sequencing, and classified it as belonging to the *Massilia* genus (Wery et al., 2003). Weon et al. and Feng et al. also used the same molecular biology approach to study *Massilia* found in other environments (Weon et al., 2009; Feng et al., 2016). It can be seen that there are many studies on *Massilia*, but its community structure composition and dynamic changes in different environments are rarely reported. This study was the first to systematically study the composition and distribution of *Massilia* spp. in constructed wetlands using the method of cloning library construction. Five pairs of primers designed in-house were innovatively used to amplify the 16S rDNA of *Massilia* spp. and further analyzed using agarose gel electrophoresis. The detection showed that the amplification effect of primer No. 5 was the best, and the electrophoresis bands of the restriction map of RFLP were abundant. Therefore, primer No. 5 was finally selected to construct a *Massilia* clone library and conduct RFLP sequence analysis. The coverage rate of the clone library constructed by primer No. 5 was greater than 86%, indicating that the clone library constructed by the primer contained most of the *Massilia spp.* in the wetland, which could more fully reflect the diversity of the *Massilia* community. The construction of the clone library can obtain a more systematic and comprehensive understanding of the community structure of *Massilia* in the constructed wetland, which provides valuable experience for studying the community composition of *Massilia* in other environments and provides data support for the construction and operation of the constructed wetland.

Previous studies have shown that the higher the H′ is, the lower the D is, and the higher the microbial diversity. The Shannon index and Simpson index of *Massilia* in the constructed wetland were analyzed, and the results showed that the variation range of Shannon index and Simpson index was 2.97–5.97 and 0.86–1.00, which were the highest in summer and significantly higher than that in winter, and the difference between spring and autumn was not significant. According to the clone library diversity index analysis, the abundance of *Massilia* in different environments of constructed wetlands in different seasons was significantly different, and the overall abundance in the summer was higher than that winter, indicating that there is a significant seasonal fluctuation in the structure and diversity of microbial communities in wetland ecosystems. When Chazarenc studied the vertical flow constructed wetland in France, he found that the degree of bacterial activity varied with seasonal temperature, the highest in June, and the lowest in late winter and autumn (Chazarenc et al., 2010), which was similar to the results of this study. This may be due to the low temperature in winter and low microbial activity, so the microbial abundance is low. According to the correlation analysis of environmental factors, T was the most significant factor affecting the community structure of *Massilia* in constructed wetlands. After the beginning of spring, with the increase of temperature, the microbial activity increases, and the reproduction speed also accelerates, so the number of microorganisms gradually increases, reaching the peak in summer. The temperature in autumn is also high, so the microorganisms continue to grow, and then the number of microorganisms starts to decrease after the temperature decreases in late autumn, until it drops to the lowest point in winter. Buckeridge et al. research found that the number of bacteria in the soil microbial community of arctic tundra decreased significantly from the peak in the late thawing period to the low point in spring, and then increased to a level similar to that in autumn in midsummer, which is different from this study (Buckeridge et al., 2013). The reason may be that the climate types and ecosystems of the two places are different. The Arctic has a long winter and freezes all the year round; Qingdao is located in the mid latitude region, with four distinct seasons and short winter. This indicates that the seasonal variation of the composition of *Massilia* in the constructed wetland may be related to the environment and climate of the area where the constructed wetland is located, and the microbial community structure of the constructed wetland in different areas is different.

This study found that *Massilia* were distributed and abundant in sewage, substrate, plant rhizosphere, phyllosphere and air in constructed wetlands, but their proportions in each environment were different. A total of 24 species of *Massilia* were detected in the constructed wetland system, and the number of species was in the following order: substrate and rhizosphere samples > sewage samples > phyllosphere samples > air samples. Moreover, the species of *Massilia* in air samples were found in other environments of the constructed wetland, indicating that the substrate (soil) and plant rhizosphere of the constructed wetland were important sources of *Massilia*. It may diffuses into the air from the underlying surface of the constructed wetland. In addition, previous studies have reported that the microbial community in the rhizosphere of constructed wetland plants is the most abundant and that plant root exudates can have a positive effect on microorganisms indicating that the constructed wetland substrate (soil) was an important source of *Massilia*. It diffuses into the air from the underlying surface of the constructed wetland (Chaparro et al., 2013; Wu et al., 2017).

Through Venn diagram comparison, we found that *Massilia* and dominant bacteria were similar in sewage samples, substrate samples, plant rhizosphere and phyllosphere samples and showed that the number of *Massilia* species in plant rhizosphere samples was slightly greater than that in substrates. This may be due to artificial wetlands using substrates, plants, microorganisms of physical, chemical and biological sewage treatment triple synergy (Stottmeister et al., 2003), and aquatic plants as an indispensable part of the purification process. This increase in the type of bacteria in constructed wetland system substrates and plant rhizospheres enhances the growth of microbial communities to create a favorable environment (Cao et al., 2017); for example, a variety of useful compounds are released into the rhizosphere (Olanrewaju et al., 2019). *Massilia* can secrete auxin, accelerate the growth of plants and increase the concentration of cations such as iron (Kuffner et al., 2010), calcium and magnesium in plant roots (Hrynkiewicz et al., 2010), improve the nutrient supply of plant roots, and improve the survival rate of plants under adversity. *Massilia* and aquatic plants complement each other, and the two act synergistically, so there are more species of *Massilia* in the rhizosphere than in the substrate.

This study found that there are 6 dominant species of *Massilia* in different environmental samples of constructed wetlands, with obvious seasonal differences, namely, *M. albidiflava, M. alkalitolerans, M. aurea, M. brevitalea, M. timonae* and *M. umbonata*. This shows that the dominant species of *Massilia* have multiple ecological functions. Among them, *M. timonae* was the most important of the six dominant bacteria, with the highest relative abundance of 58.75%. Because of its significant positive correlation with T, the relative abundance of *M. timonae* was higher in summer and autumn, and lower in winter and spring. In 2009, Faramarzi et al. found that the most dominant species, *M. timonae,* could produce chitinase in a medium containing colloidal chitin as the only carbon and nitrogen source, which greatly reduced the production cost of chitinase (Faramarzi et al., 2009). The relative abundance of the second largest species *M. brevitalea* was higher in winter and spring, reaching 54.76%. The correlation analysis of environmental factors showed that it was significantly negatively correlated with T, so it was a low temperature resistant *Massilia*. In 2008, Zul et al. isolated a strain of *M. brevitalea* from the substrate and found that it can hydrolyze starch and casein and has salt tolerance, and in this study, it was found that its relative abundance was high in the winter (Zul et al., 2008). In addition, *M. brevitalea* was once isolated from organic fertilizers as the dominant strain of ammonification, indicating that it is more suitable for survival in environments with higher organic matter and nitrogen content, and its abundance was positively correlated with total nitrogen and organic matter. There is little difference between *M. alkalitolerans* and *M. aurea*, but *M. alkalitolerans* is slightly dominant, and the relative abundance can reach 44.12%, while the highest relative abundance of *M. aurea* is 17.24%. *M. alkalitolerans* has alkali resistance (Xu et al., 2005), and *M. aurea* can produce auxin (indoleacetic acid) (Gallego et al., 2006). The relative abundances of *M. albidiflava* and *M. umbonata* were low. *M. umbonata* can produce poly-β-hydroxybutyrate (Rodriguez-Diaz et al., 2014). *M. albidiflava* was positively correlated with total nitrogen, which may be related to its nitrogen fixation activity (Zhang et al., 2006).

In addition, the environmental factors in the constructed wetland have a significant impact on the composition, distribution and seasonal variation of the community structure of the *Massilia* dominant bacteria. In this study, we found that *M. albidiflava* and *M. brevitalea*, the dominant species of *Massilia*, were positively correlated with NH_3_-N, NO_2_^−^-N and NO_3_^−^-N, consistent with their having nitrate reductase activity; *M. timonae* and *M. aurea* were positively correlated with TP content, which proved that *Massilia* had phosphorus solubilization; *M. aurea* absorbed the fungal secretion; The photosynthetic products secreted by the fungus were absorbed by *M. aurea*, which promoted the mineralization and transformation of organic phosphorus in the substrate. The substrate with high phosphorus content was more suitable for its survival (Wang et al., 2016). When studying the correlation between microbial community structure and environmental factors in Poyang Lake wetland profile, it was found that water content and pH were the main factors affecting bacterial community structure (Ma et al., 2018), which was consistent with the results of this study. Since airborne bacteria are usually attached to the surface of atmospheric particles and can easily spread with the wind. Many air environment factors affect atmospheric particulate matter, which in turn affects the composition and function of the microbial community attached to its surface (Hwang and Park, 2014). Therefore, the air environmental factors investigated in this study, such as temperature, humidity, wind speed, PM_2.5_ and PM_10_, all had an impact on the seasonal variation and community structure of the *Massilia* dominant strains (Maron et al., 2006; Cao et al., 2014). In summary, the study of *Massilia* dominant species in the constructed wetland will help us to analyze the main functions of the constructed wetland and adjust its operation status, which can make it treat sewage more efficiently.

Although the genus *Massilia* has multiple environmental functions, clinical medical studies have found that infection with one of the dominant species, *M. timonae*, has the potential to lead to multiple human health-threatening diseases, such as septicemia, osteomyelitis, and encephalitis (Scola et al., 1998; Van Craenenbroeck et al., 2011), but the infection mechanism is unknown. Song recently isolated a *Massilia oculi* sp. nov. of type strain CCUG 43427^T^ from the eyes of an endophthalmitis patient, and analyzed its genome. It was found that it contains pathogenic genes, indicating that the strain has posed a threat to human health (Song et al., 2019). The above results indicate that some *Massilia* bacteria have evolved a wide range of pathogenicity, and the air mosaic bacteria in constructed wetlands can spread with the air to the surrounding environment, which is one of the important ways of *Massilia* transmission. Therefore, studying the community composition of *Massilia* bacteria is important for the safety evaluation of artificial wetlands, as well as for public health and ecological safety.

## 5. Conclusion

In conclusion, self-designed primers were used to construct a 16S rDNA clone library, and RFLP sequence analysis was used to determine the composition of the *Massilia* community in sewage, substrate, plant rhizosphere, plant leaf and air in an artificial wetland sewage treatment system. The overall community of *Massilia* bacteria in the artificial wetland sewage treatment system was higher in the summer and autumn than in the spring and winter, while the relative abundance of the dominant species had obvious seasonal differences. There were significant seasonal differences in the relative abundance of dominant species in sewage, substrate, plant rhizosphere and plant leaf sphere. Environmental factors in the sewage, substrate and air of the constructed wetland had a great influence on the composition and seasonal variation of *Massilia’s* community structure. The diversity of *Massilia* bacteria in the artificial wetland air was less different than in the environmental samples, and the overlap rate of the total OTUs with the remaining samples was 100%, indicating that *Massilia* spp. in the air were probably from the underlying surface of the constructed wetland. In different seasons, the composition of *Massilia* spp. was significantly different in the same environment. In the same season, the similarity of *Massilia* spp. in different environments was high, and there was homology.

## Data availability statement

The original contributions presented in the study are included in the article/supplementary material, further inquiries can be directed to the corresponding author.

## Author contributions

AX wrote the review and editing. CL wrote the original draft preparation. All authors contributed to the article and approved the submitted version.

## Funding

This research was funded by the Key R&D projects of Shandong Province (2018GSF117022), the Youth Foundation of Shandong Natural Science Foundation (ZR2020QC027), and the Shandong Natural Science Foundation (ZR2023MC193).

## Conflict of interest

The authors declare that the research was conducted in the absence of any commercial or financial relationships that could be construed as a potential conflict of interest.

## Publisher’s note

All claims expressed in this article are solely those of the authors and do not necessarily represent those of their affiliated organizations, or those of the publisher, the editors and the reviewers. Any product that may be evaluated in this article, or claim that may be made by its manufacturer, is not guaranteed or endorsed by the publisher.
